# Re-testing and misclassification of HIV-2 and HIV-1&2 dually reactive patients among the HIV-2 cohort of The West African Database to evaluate AIDS collaboration

**DOI:** 10.7448/IAS.17.1.19064

**Published:** 2014-08-12

**Authors:** Boris K Tchounga, Andre Inwoley, Patrick A Coffie, Daouda Minta, Eugene Messou, Guillaume Bado, Albert Minga, Denise Hawerlander, Coumba Kane, Serge P Eholie, François Dabis, Didier K Ekouevi, for the WADA Collaboration

**Affiliations:** 1 Centre INSERM U897-Epidémiologie-Biostatistique, ISPED, Université de Bordeaux, Bordeaux, France; 2 Inserm U897, ISPED, Université de Bordeaux, Bordeaux, France; 3 Programme PACCI, Site de recherche ANRS, Abidjan, Côte d’Ivoire; 4 Centre de Diagnostic et de Recherche sur le SIDA et les Affections Opportunistes, CHU de Treichville, Abidjan, Côte d’Ivoire; 5 Service des Maladies Infectieuses et Tropicales, CHU de Treichville, Abidjan, Côte d’Ivoire; 6 Centre de Prise en Charge des Personnes vivant avec le VIH, Hôpital du Point G, Bamako, Mali; 7 Centre de Prise en Charge de Recherche et de Formation, CePReF-Aconda-VS, Abidjan, Côte d’Ivoire; 8 Hôpital de Jour, Service des Maladies Infectieuses et Tropicales, CHU Souro Sanou, Bobo Dioulasso, Burkina Faso; 9 Centre Médical de Suivi de Donneurs de Sang/CNTS/PRIMO-CI, Abidjan, Côte d’Ivoire; 10 Centre Intégré de Recherches Biocliniques d’Abidjan CIRBA, Abidjan, Côte d’Ivoire; 11 Laboratoire de Bactériologie-Virologie, Département GC&BA-ESP/UCAD, CHU A Le Dantec, Dakar, Sénégal; 12 Département des Sciences Fondamentales et Santé Publique, Université de Lomé, Lomé-Togo

**Keywords:** HIV-2, HIV-1&2 dually reactive, testing, classification, West Africa

## Abstract

**Introduction:**

West Africa is characterized by the circulation of HIV-1 and HIV-2. The laboratory diagnosis of these two infections as well as the choice of a first-line antiretroviral therapy (ART) is challenging, considering the limited access to second-line regimens. This study aimed at confirming the classification of HIV-2 and HIV-1&2 dually reactive patients followed up in the HIV-2 cohort of the West African Database to evaluate AIDS collaboration.

**Method:**

A cross-sectional survey was conducted from March to December 2012 in Burkina Faso, Côte d’Ivoire and Mali among patients classified as HIV-2 or HIV-1&2 dually reactive according to the national HIV testing algorithms. A 5-ml blood sample was collected from each patient and tested in a single reference laboratory in Côte d’Ivoire (CeDReS, Abidjan) with two immuno-enzymatic tests: ImmunoCombII^®^ (HIV-1&2 ImmunoComb BiSpot – Alere) and an in-house ELISA test, approved by the French National AIDS and hepatitis Research Agency (ANRS).

**Results:**

A total of 547 patients were included; 57% of them were initially classified as HIV-2 and 43% as HIV-1&2 dually reactive. Half of the patients had CD4≥500 cells/mm^3^ and 68.6% were on ART. Of the 312 patients initially classified as HIV-2, 267 (85.7%) were confirmed as HIV-2 with ImmunoCombII^®^ and in-house ELISA while 16 (5.1%) and 9 (2.9%) were reclassified as HIV-1 and HIV-1&2, respectively (Kappa=0.69; *p*<0.001). Among the 235 patients initially classified as HIV-1&2 dually reactive, only 54 (23.0%) were confirmed as dually reactive with ImmunoCombII^®^ and in-house ELISA, while 103 (43.8%) and 33 (14.0%) were reclassified as HIV-1 and HIV-2 mono-infected, respectively (kappa= 0.70; *p*<0.001). Overall, 300 samples (54.8%) were concordantly classified as HIV-2, 63 (11.5%) as HIV-1&2 dually reactive and 119 (21.8%) as HIV-1 (kappa=0.79; *p*<0.001). The two tests gave discordant results for 65 samples (11.9%).

**Conclusions:**

Patients with HIV-2 mono-infection are correctly discriminated by the national algorithms used in West African countries. HIV-1&2 dually reactive patients should be systematically investigated, with a standardized algorithm using more accurate tests, before initiating ART as at least 4 out of 10 of them could initiate an effective first-line ART for HIV-1 and optimize their second-line treatment options.

## Introduction

West Africa is characterized by the circulation of both HIV-1 and HIV-2, which leads to co-infections with HIV-1 and HIV-2 (HIV-1&2) [1–4]. The biological diagnosis of these co-infections [5–7] as well as the choice of a first-line antiretroviral therapy (ART) is still challenging, when considering the natural resistance of HIV-2 to non-nucleoside reverse transcriptase inhibitors (NNRTIs) [8, 9] and the limited access to second and third-line ART in low- and middle-income countries [10–13].

Several algorithms have been adopted for the routine diagnosis of HIV infection about 15 years ago in most West African countries, in line with the US Centres for Disease Control and prevention (CDC) and World Health Organization (WHO) recommendations [14]. Most of them were based on the serial use of two rapid serological tests at the peripheral level, with a third immuno-enzymatic test in case of discordance [7, 15]. As HIV-2 is resistant to NNRTIs [8, 9], and as viral resistance to first and second-line ART has emerged [16–19], the choice of ART for HIV-2 differs from that for HIV-1. It is therefore mandatory to discriminate well between HIV types before initiating ART in West Africa [20].

The national algorithms of many West African countries are thus based on serological tests allowing the simultaneous detection of HIV-1- and HIV-2-specific antibodies [7, 15]. However, many studies have reported the difficulties of these algorithms to accurately discriminate between patients exclusively infected with HIV-2 and patients dually infected with HIV-1 and HIV-2 [7].

The HIV-2 West Africa cohort is composed of 4050 HIV-2 and HIV-1&2 dually seropositive patients. It is embedded in the West African Database to evaluate AIDS Collaboration (WADA), which is part of the International epidemiological Database to Evaluate AIDS (IeDEA) network [21]. In brief, 13 clinics in 5 countries (Benin, Burkina-Faso, Côte d’Ivoire, Mali and Senegal) are contributing to the West Africa HIV-2 cohort [22]. Patients are included in this cohort based on the results of HIV testing performed at clinical sites according to the national algorithms of each participating country. In order to validate the diagnosis and initial classification of patients of the WADA HIV-2 cohort, a re-testing was proposed to a panel of participants. Here, we describe the results of this retesting organized in three West African countries.

## Method

### Study design

A cross-sectional survey was conducted from March to December 2012 in Burkina Faso, Côte d’Ivoire and Mali among patients classified as HIV-2 and HIV-1&2 dually reactive, and followed up in the clinical sites of the WADA HIV-2 cohort.

### Study sample

All patients aged 18 years and above, registered in the WADA HIV-2 database, who attended one of the participating clinics during the study period were invited to participate in this survey regardless of ART initiation.

### Data collection

A standardized survey form was used to collect data about patients’ demographics, their clinical and biological characteristics from the enrolment in the cohort till the time of the study. The main assays used for initial HIV diagnosis were Determine^®^HIV-1/2 (Abbott Laboratories; sensitivity 100%; specificity 99.4%), GenieII^®^HIV1/HIV2 (Bio-Rad; sensitivity 100%; specificity 99.7%), SDBioline^®^HIV 1/2 3.0 (Standard diagnostics. sensitivity 100%; specificity 99.3%) [23].

### Biological retesting

A total of 5 ml of blood was collected from each patient and sent to the referral laboratory of the study (CeDRes Abidjan). These samples were then tested for the detection of HIV-2 or HIV-1&2 dual infection using two different tests performed independently. The first one was ImmunoCombII^®^ (ImmunoComb HIV-1&2-BiSpot Alere/Orgenics), a WHO-endorsed, indirect, immuno-enzymatic test (sensibility 100%; specificity 99.7%) [23]. This test allows the detection of antibodies against glycoproteins of HIV-1 and HIV-2. The second test was an in-house, indirect immuno-enzymatic test allowing the qualitative detection of both HIV-1 and HIV-2 antibodies. This test has been adapted from an assay developed for HIV-1 by Barin *et al*. [24], and has shown 98% specificity [25] and 80% concordance with real-time Deoxyribonucleic acid (DNA) Polymerase chain reaction (PCR) [7]. It is approved by the French National AIDS and Hepatitis Research Agency (ANRS) and routinely used in the reference laboratory of the study as home-made ELISA. A real-time PCR assay developed and validated by the ANRS [26, 27] was further used to detect plasma HIV-1 Ribonucleic acid (RNA) in samples confirmed as dually reactive for HIV-1 and HIV-2 after retesting.

### Data analysis

A data analysis was performed using Stata software (StataTM 9.0 College Station, Texas, USA). The Chi-square test or the Fisher exact test was used to compare proportions while the non-parametric test of Kruskall–Wallis was used for the comparison of median values and distributions of quantitative variables. The kappa statistic was use to explore the concordance between the laboratory tests.

### Ethics

This survey was approved by the national ethics committee of each participating country. Patients were informed and had to give their written consent before being included.

## Results

### Demographic and follow-up characteristics

A total of 547 patients were enrolled in this study, 312 (57.0%) were HIV-2 infected and 235 (43.0%) were HIV-1&2 dually reactive according to the national algorithms used in each participating country: 232 (42.4%) were from Burkina Faso, 268 (49.0%) were from Côte d’Ivoire and 47 (8.6%) were from Mali. Majority were women (*n*=337, 61.6%) and the median age was 47 years (inter-quartile range [IQR] [40–53]). Table 1 presents the main baseline and follow-up characteristics of the study sample.

**Table 1:** Baseline and follow-up characteristics of patients according to initial HIV status in a sample of the WADA HIV-2 cohort the surveyed in 2012

	HIV status according to national algorithms					
	
	HIV-2		HIV-1&2		Total	
			
	*N*=312	%	*N*=235	%	*N*=547	%
Age (years)						
Median (IQR)	48 [42–54]		46 [38–52]		47 [40–53]	
Gender						
Female	178	57.1	159	67.7	337	61.6
Years of HIV diagnosis						
<2006	46	14.7	29	12.3	75	13.7
2006–2009	47	15.1	19	8.1	66	12.1
2009–2012	219	70.2	187	79.5	406	74.2
Country						
Burkina Faso	92	29.5	140	59.6	232	42.4
Cote d’Ivoire	194	62.2	74	31.5	268	49.0
Mali	26	8.3	21	8.9	47	8.6
CD4 (cells/µL) at inclusion in the cohort						
<350	117	37.5	158	67.2	275	50.3
350–500	79	27.3	40	17.0	119	21.7
≥500	116	35.2	37	15.8	153	28.0
CD4 (cells/µL) at enrolment in the study						
<350	81	26.0	67	28.5	148	27.1
350–500	64	20.5	53	22.5	117	21.4
≥500	167	53.5	115	49.0	282	51.5
ART initiation						
Yes	174	55.8	201	85.5	375	68.6

ART=antiretroviral treatment; IQR=inter-quartile range; WADA=West African database to evaluate AIDS; HIV-2=human immunodeficiency virus type 2; HIV-1&2=human immunodeficiency virus type 1 and type 2.

Half of the patients enrolled (50.3%) had CD4 counts <350 cells/micro litre (cell/µL) and 119 (21.7%) had CD4 counts between 350 and 500 cell/µL at enrolment in the cohort. The overall median follow-up was 3.2 years [IQR 1.2–6.3]. More than two-thirds of the patients (68.6%) were on ART at the time of the survey; among them, 301 (83.3%) had initiated a regimen composed of two nucleoside reverse transcriptase inhibitors (NRTIs) and a protease inhibitor (PI), 51 (13.6%) had started ART with two NRTIs+one NNRTI and seven (1.8%) had initiated a three-NRTI-based regimen. At the time of blood sample collection, the overall median CD4 count was 482 cells/µL [IQR 312–656], and more than half of the patients (51.5%) had CD4 counts ≥500 cells/µL.

### HIV testing and differentiation on clinical site

The diagnosis of HIV-2 infection and HIV1&2-dual infection was made between 2009 and 2012 in 406 patients (74.2%) and before 2009 in the 141 remaining ones. Data on the serological tests used for HIV diagnosis in clinical sites were reported only for 373 patients (68.2%). Determine^®^ was used as the first test in the three countries. GenieII^®^ was primarily used as the second test of the series in Côte d’Ivoire (66.8%) and in Mali (50.0%), while in Burkina Faso it was SD Bioline^®^ (63.8%).

### Results of serological retesting

Overall, between the 547 samples retested with ImmunoCombII^®^ and home-made ELISA, 300 (54.8%) were concordantly classified as HIV-2, 63 (11.5%) as HIV-1&2 dually reactive and 119 (21.8%) as HIV-1 (kappa= 0.79; *p*<0.001) (Table 2). Both tests gave discordant results for 65 samples (11.9%).

**Table 2:** Results of the retesting of HIV-2 and HIV-1&2 dually reactive patients with two serological tests in the WADA HIV-2 cohort in 2012

	Status on site		ImmunoComb^®^		Home-made ELISA		Concordant^a^ results with both tests	
				
	*N*	%	*N*	%	*N*	%	*N*	%
HIV-1	0	0	125	22.9	145	26.5	119	21.8
HIV-2	312	57.0	303	55.4	331	60.5	300	54.8
HIV-1&2	235	43.0	119	21.8	71	13.0	63	11.5
Total	547	100	547	100	547	100	482	88.1

a Positive–positive or negative–negative. WADA=West African database to evaluate AIDS; HIV-1=human immunodeficiency virus type 1; HIV-2=human immunodeficiency virus type 2; HIV-1&2=human immunodeficiency virus type 1 and type 2.

Of the 312 patients initially classified as HIV-2, 267 (85.7%) were confirmed as HIV-2 with ImmunoCombII^®^ and In house ELISA tests, while 16 (5.1%) and 9 (2.9%) were reclassified as HIV-1 and HIV-1&2 respectively (Kappa=0.69; *p*<0.001). Both tests gave discordant results for 20 patients (6.4%), (Table 3).

Among the 235 patients initially classified as HIV-1&2 dually reactive, only 54 (23.0%) were confirmed as dually reactive with ImmunoCombII^®^ and In house ELISA, while 103 (43.8%) and 33 (14.0%) were reclassified as HIV-1- and HIV-2 mono-infected respectively (kappa=0.70; *p*<0.001). Both tests gave discordant results for 45 patients (19.1%), (Table 3).

**Table 3:** Results of the retesting in a sample of the WADA HIV-2 cohort in 2012

312 patients initially classified as HIV-2 infected									

		In-house ELISA							
		
		HIV-1		HIV-2		HIV-1/2		Total	
					
	ImmunoCombII^®^	*N*	%	*N*	%	*N*	%	*N*	%
**HIV-2 on site**	HIV-1	**16**	**5.1**	0	0	1	0.3	17	5.4
	HIV-2	0	0	**267**	**85.7**	2	0.6	269	86.3
	HIV-1/2	1	0.3	**16**	5.1	**9**	**2.9**	26	8.3
	Total	17	5.4	283	90.7	12	3.8	**312**	**100**
**235 patients initially classified as HIV-1&2 dually**									

		**In-house ELISA**							
		
		**HIV-1**		**HIV-2**		**HIV-1/2**		**Total**	
					
	**ImmunoCombII** ^®^	***N***	**%**	***N***	**%**	***N***	**%**	***N***	**%**

**HIV-1/2 on site**	HIV-1	**103**	**43.8**	0	0	5	2.1	108	46.0
	HIV-2	1	0.4	**33**	**14.0**	0	0	34	14.5
	HIV-1/2	24	10.1	15	6.4	**54**	**23.0**	93	39.5
	Total	128	54.5	48	20.4	59	25.1	**235**	**100**

Bold values indicate that both confirmatory tests are concordant. The grey box indicates full agreement with initial screening test results. WADA=IeDEA West African database to evaluate AIDS; HIV-1=human immunodeficiency virus type 1; HIV-2=human immunodeficiency virus type 2; HIV-1&2=human immunodeficiency virus type 1 and type 2.

### PCR HIV-1 RNA detection

Plasma viral load was available for 54 of the 63 patients confirmed to be HIV-1&2 dually reactive with the two tests; 40 (74.0%) were further tested with a real-time PCR technique for the detection of HIV-1 RNA. Among them, 36 (90%) were on treatment. HIV-1 RNA was detected in three patients (7.5%) and among them, two were on treatment.

## Discussion

Within WADA HIV-2 cohort, 547 patients initially classified as HIV-2 infected (57%) or HIV-1&2 dually reactive (43%) were retested using ImmunoCombII^®^ and an in-house ELISA performed independently. Overall, the diagnosis of HIV-2 mono-infection was confirmed by the two tests for more than 85% of those originally considered HIV-2 infected; in addition, 14% of the study subjects originally considered as HIV-1&2 were reclassified as HIV-2 only. Conversely, the diagnosis of HIV-1&2 dual reactivity was confirmed by these two tests only in almost one case out of five (23%), while more than 43% of patients initially HIV-1&2 dually reactive were reclassified as HIV-1 mono-infected.

Our results suggest that as HIV-1 mono-infection, HIV-2 mono-infection is correctly diagnosed at clinical sites in most instances (267/300, 89%) with the rapid serological tests proposed by the national algorithms. However, it remains challenging to clearly make the difference between the HIV-1&2 dually infected patients and the mono-infected ones. These results are consistent with previous reports [5, 6], including that of Rouet *et al*. who reported the difficulty to confirm the dual infection with HIV-1 and HIV-2 in a population of pregnant women in Abidjan [7]. Currently, HIV screening strategies in France [28] and in the United States [29, 30] are based on algorithms using a fourth generation assay which accurately detects early HIV infection but is unable to differentiate between HIV-1 and HIV-2. A Western Blot or a Multispot rapid HIV-1/HIV-2 differentiation assay is then used for the differentiation and in case of discordance a supplemental nucleic acid test (HIV-1 RNA) is performed [31]. Although these algorithms have shown higher diagnostic accuracy for the early detection and the differentiation between HIV-1 and HIV-2, none of them are currently considered as the gold standard for the diagnosis of HIV-1&2-dual infection. It is therefore critical to develop new serological tests that are more discriminatory and specific to HIV-1&2-dual infection. This would improve the algorithms accuracy for HIV diagnosis and discrimination in countries where both HIV-1 and HIV-2 are endemic and could help improving ART management [20].

The recent 2013 WHO guidelines recommend initiating lopinavir/ritonavir plus two NRTIs as the preferred first-line ART option for HIV-2 and HIV-1&2 dually infected patients, since HIV-2 is intrinsically resistant to NNRTIs [20]. Our study reveals that 43.8% of patients initially considered HIV-1&2 dually reactive and 5.1% of those initially considered HIV-2 infected are in fact HIV-1 mono-infected. Thus, the frequent misdiagnosis of HIV-1&2 dually reactive patients leads to prematurely initiate a preferred second-line ART in many HIV-1 infected patients, in areas with limited access to treatment options. Thus, it is critical to improve the diagnosis and classification of HIV-2 and HIV-1&2 dually infected patients before initiating ART, in order to preserve second- and upper-line ART options in the West African region [32].

The detection of HIV-1 and HIV-2 RNA or DNA using type-specific PCR for the isolation of both viruses from the same individual could be a solution to this problem [33–35]. However, HIV 2 RNA is undetectable in about 40% of infected individuals before treatment initiation in Europe and in West Africa [36, 37]. RNA tests cannot therefore be used as a diagnostic test of HIV-2 infection. Furthermore, no commercial test is available right now for the detection of HIV-2 DNA. Although many in-house techniques are being developed, their implementation in resource-constrained settings will be challenging [37, 38]. The clinical research consequence is that it remains very difficult in practice to characterize a cohort of HIV-1&2 dually infected patients. Since our study reveals that about 77% of the HIV-1&2 dually reactive patients are misclassified according to national algorithms, the published cohort findings on ART response should be taken with caution, especially when molecular biology techniques have not been used for the confirmation [39, 40].

This is the first international survey retesting more than 500 HIV-2 and HIV-1&2 dually reactive patients in three West African countries. The lack of an internationally recognized gold standard for the discrimination of HIV-2 and HIV-1&2 dually infected patients did not allow us to propose and evaluate a new testing algorithm for the peripheral clinical sites. Moreover, considering the usually low levels of HIV-2 RNA viremia and the difficulties to implement DNA detection techniques in resource-constrained settings, specific research is needed to improve the current algorithms.

## Conclusions

Most HIV-2 mono-infected patients are correctly discriminated by the national algorithms currently used in West African countries. HIV-1&2 dually reactive patients should be systematically retested with more accurate serological tests such as those used in this study or with DNA testing when available, before ART initiation. This would better prepare the use of second-line ART regimens when needed, especially in this part of the world.
